# Electrical and Optical Properties of Nb-doped SrSnO_3_ Epitaxial Films Deposited by Pulsed Laser Deposition

**DOI:** 10.1186/s11671-020-03390-1

**Published:** 2020-08-17

**Authors:** Kaifeng Li, Qiang Gao, Li Zhao, Qinzhuang Liu

**Affiliations:** 1 School of Physics and Electronic Information, Huaibei Normal University, Huaibei, 235000 People’s Republic of China; 2 Anhui Province Key Laboratory of Pollutant Sensitive Materials and Environmental Remediation, Huaibei Normal University, Huaibei, 235000 People’s Republic of China

**Keywords:** SrSnO_3_, Thin films, Pulsed laser deposition, TCO, Epitaxial, Oxygen vacancies

## Abstract

Nb-doped SrSnO_3_ (SSNO) thin films were epitaxially grown on LaAlO_3_(001) single-crystal substrates using pulsed laser deposition under various oxygen pressures and substrate temperatures. The crystalline structure, electrical, and optical properties of the films were investigated in detail. X-ray diffraction results show that the cell volume of the films reduces gradually with increasing oxygen pressure while preserving the epitaxial characteristic. X-ray photoelectron spectroscopy analysis confirms the Nb^5+^ oxidation state in the SSNO films. Hall-effect measurements were performed and the film prepared at 0.2 Pa with the 780 °C substrate temperature exhibits the lowest room-temperature resistivity of 31.3 mΩcm and Hall mobility of 3.31 cm^2^/Vs with a carrier concentration at 6.03 × 10^19^/cm^3^. Temperature-dependent resistivity of this sample displays metal-semiconductor transition and is explained mainly by electron-electron effects. Optical transparency of the films is more than 70% in the wavelength range from 600 to 1800 nm. The band gaps increase from 4.35 to 4.90 eV for the indirect gap and 4.82 to 5.29 eV for the direct by lowering oxygen pressure from 20 to 1 × 10^−3^ Pa, which can be interpreted by Burstein-Moss effect and oxygen vacancies generated in the high vacuum.

## Background

Transparent conducting oxides (TCO) are extraordinary materials that have both low electrical resistivity and high optical transparency. The combination of the two significant features makes TCO key materials for wide applications in optoelectronic devices, such as *pn* junctions, field-effect transistors, and solar cells [1–7]. As the typical TCO material, Sn-doped In_2_O_3_ has been widely used due to its high transmittance of more than 90% in the visible spectral region and excellent conductivity of 1 × 10^4^ S/cm [8]. Other well-known TCOs, including Al-doped ZnO [9, 10] and Sb-doped SnO_2_ [11], are also of interest. These rather high-performance conductive characteristics are correlated with the special electronic structure, in which the conduction band is consisted of delocalized metallic *s* orbitals, leading to low electron effective mass and high dispersion. In a given material, low effective mass and high dispersion can result in high electrical conductivity. However, these binary oxides have their own limitations such as the thermal and chemical instability, which will cause degradation problems for the operation of oxide electronic applications. Therefore, there are a lot of efforts for finding alternative materials with satisfactory performance to compensate the weaknesses of binary oxides. Materials with the perovskite structure have been discovered to exhibit exceptional physical properties, such as superconductivity [12], multiferroicity [13], and colossal magnetoresistance [14]. Compared with the conventional binary TCOs, they show better structural stability and can be more flexible in chemical modification, which may improve their physical properties or contribute to realizing the novel functionalities.

Alkaline-earth stannates *A*SnO_3_ (*A* = Ca, Sr, and Ba) are of great interest due to their intriguing dielectric properties and applications such as in thermally stable capacitors [15–17]. Recently, these perovskites have also received considerable attentions as new TCO films based on the unique features of high optical transparency and high carrier mobility [18–22]. Their valence bands consist mainly of O 2*p* orbitals and the conduction bands are largely contributed by Sn 5*s* orbitals located above the Fermi level, making wide band gaps [23]. The small electron effective masses, thus, good electrical conductivity of *A*SnO_3_ can be ascribed to the large size of Sn that gives the conduction band edge with antibonding *s* characters [23]. Among *A*SnO_3_, SrSnO_3_ (SSO) exhibits the semiconductor behavior with the band gap of 4.1 eV and has an orthorhombic structure with the lattice constants of *a* = 5.708 Å, *b* = 5.703 Å, and *c* = 8.065 Å [24]. The pure SSO films are not conductive. To further improve the conductivity of SSO films, many elements have been chosen to dope SSO films to generate carriers by replacing the A site or B site, such as La for Sr sites [25], and Ta for Sn sites [26]. Selecting proper doping element and appropriate dopant concentration is crucial to obtain high-performance TCO films, as well as optimized growth condition. Nb element is often adopted to substitute partially for TCO films, as more carriers tend to generate in the material system benefiting from the high valence-state Nb^5+^ cations. Indeed, the high density of carriers created by introducing Nb ions has been demonstrated according to the experimental and first-principle calculation results. For examples, Nb-doped TiO_2_ films exhibited the room-temperature resistivity as low as 2 × 10^−4^ Ωcm [27], and Nb-doped ZnO films showed the lowest resistivity of 8.95 × 10^−4^ Ωcm [28]. From the viewpoint of intrinsic physics and basic science, the Nb donors will donate one electron to the conduction band of SSO as the Sn ions are substituted by Nb due to the similar ionic radii of Nb^5+^ (0.64 Å) and Sn^4+^ (0.69 Å), and will result in an enhanced conductivity in electron-doped SSO films. On the other hand, the physical properties of TCO films can also be tuned under different deposition oxygen pressures and temperatures during the film growth. Transport performances of BaSnO_3_ (BSO) [29], LaNiO_3_ [30], and SrTiO_3_ films [31], and the magnetic behavior of the Gd-doped ZnO films [32, 33] were previously reported to show sensitivity to the oxygen pressure. La-doped SSO films and Nd-doped BSO films also exhibited a remarkable correlation between electrical properties and deposition temperature [20, 34]. To better control the physical performances and to obtain high-quality thin films for investigation, it has great significance to study the impacts of these key deposition conditions on the thin films. However, previous reports are very limited on the effects of oxygen pressure and substrate temperature on the structural, optical, and electrical properties of SSO thin films. Therefore, we focus on this aspect of this work. A series of Nb-doped SSO films were fabricated by pulsed laser deposition (PLD). To avoid influencing the transmittance measurements of the films, the LaAlO_3_ single-crystal substrates with a wide band gap of 5.5 eV were employed to deposit the thin films. The oxygen pressure during growth varies from 1 × 10^−3^ to 20 Pa and the substrate temperature from 660 to 820 °C. The structure, electrical, and optical properties of the films were investigated in detail.

## Methods

Sr(Sn_0.95_Nb_0.05_)O_3_ (SSNO) target was fabricated by solid-state reactions using high purity SrCO_3_, SnO_2_, and Nb_2_O_5_ as raw materials with the final sintering temperature kept at 1520 °C for 10 h. Two groups of epitaxial SSNO thin films (A and B) were grown on LaAlO_3_(001) [LAO(001)] substrates by PLD employing a 248 nm KrF excimer laser to ablate the SSNO target with a repetition rate of 3 Hz. The laser energy density on the rotating surface of the target was about 1.8 J/cm^2^, and the substrate-to-target distance was kept at 55 mm. Samples of group A were first prepared to optimize the resistivity with varying oxygen pressure from 1 × 10^−3^ to 20 Pa while keeping the substrate temperature constant at 780 °C. Based on the optimized oxygen pressure, films in group B were then deposited at different substrate temperatures between 660 and 820 °C to further explore the optimal growth condition. Prior to flowing pure oxygen, the base pressure of the chamber was 1 × 10^−4^ Pa. After deposition, all the films were annealed in situ for 15 min before being cooled down in the same oxygen ambient. Film structures were characterized using high-resolution X-ray diffraction (XRD) from diffractometer Empyrean PANalytical with a Cu K*α*_1_ source (*λ* = 1.5406 Å). In-plane and out-of-plane diffraction 2*θ*-*ω* scans and *φ* scans were carried out to determine the epitaxial growth. *ω* scans were performed to confirm the crystallinity of the films, and reciprocal space mappings (RSMs) were employed to investigate the strain state. Growth rates of the films were estimated using X-ray reflectivity, and the thickness of each deposited film was controlled at 230 nm. X-ray photoelectron spectroscopy (XPS, Thermo, escalab 250XI) was used to analyze the valence states of the elements. Hall-effect measurements were performed using van der Pauw geometry on an Ecopia HMS-3000 system at room temperature. Curves of the temperature dependence of resistivity for the films were obtained in the temperature range from 300 to 30 K using the standard four-terminal method with a Keithley 2400 source meter. The optical transmittance in the wavelength range of 200-1800 nm was measured by UV-vis spectrometer (Lambda 950, Perkin Elmer, USA).

## Results and Discussion

**Fig. 1:**
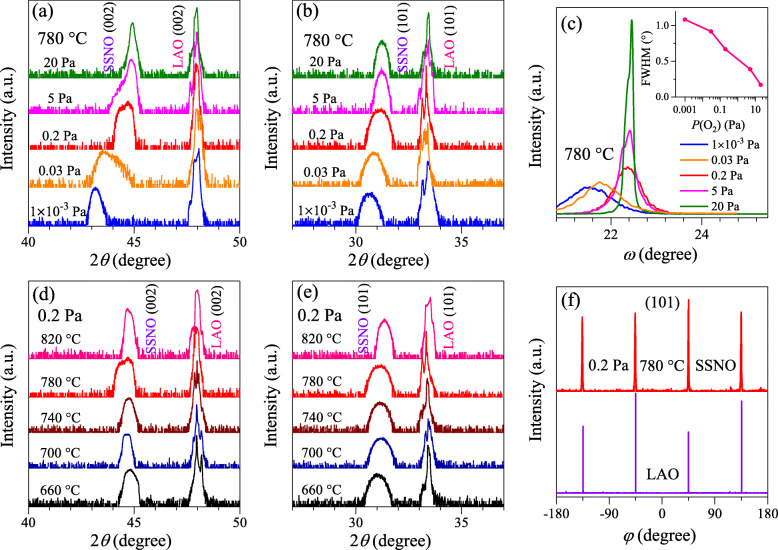
XRD 2*θ*-*ω* scan patterns of the SSNO films on LAO(001) substrates along **a** (002) and **b** (101) orientations with different oxygen pressures. **c** XRD *ω*-rocking curves taken on the (002) peaks of the films. The inset shows the FWHM results by varying oxygen pressure from 1 × 10^−3^ to 20 Pa. **d** and **e** are the 2*θ*-*ω* scans of the films with various substrate temperatures along (002) and (101) planes, respectively. **f** *φ* scans of the SSNO film deposited at 0.2 Pa and 780 °C and LAO substrate around (101) reflections

Figure 1a and b show the 2*θ*-*ω* linear scans of the SSNO films with various oxygen pressures grown on LAO(001) single crystalline substrates along (002) and (101) orientations, respectively. With the substrate temperature kept at 780 °C, the films in group A were fabricated under 1 × 10^−3^, 0.03, 0.2, 5, and 20 Pa, as denoted. Only the (002) and (101) reflection peaks can be observed, indicating that there is no other impurity phase in the films. It can be further proved by the wide-ranged XRD 2*θ*-*ω* scan of the film prepared at 20 Pa and 780 °C, as a representative as shown in Fig. S1. To investigate the effect of Nb dopants on the structural properties, the XRD result of the SSO film with the same growth condition is also presented in Fig. S1. It is observed that the (00 *l*) peaks of the SSNO film move to higher 2*θ* angles in comparison to those of the SSO film, which can be attributed to the difference in the ionic radii of Nb^5+^ (0.64 Å) and Sn^4+^ (0.69 Å). With decreasing deposition oxygen pressure in group A, the diffraction peaks move to lower 2*θ* angles gradually, giving an increase in lattice parameters and cell volume. Figure 1c presents the rocking curves taken from (002) peaks of the SSNO films in group A. The full width at half maximum (FWHM) as a function of oxygen pressure is shown in the inset of Fig. 1c. As the oxygen pressure increases from 1 × 10^−3^ to 20 Pa, the FWHM value decreases from 1.08° to 0.17°, indicating that the film prepared at 20 Pa possesses the highest crystallinity. It is noticed that more oxygen is beneficial for the nucleation, crystallization, and growth of SSNO films. Similar phenomenon has been reported in oxygen-deficient SSO films and ZnO films [35, 36]. Figure 1d and e depict the SSNO films in group B oriented along (002) and (101) reflections without other phases. These samples were deposited at 0.2 Pa with substrate temperatures of 660, 700, 740, 780, and 820 °C. XRD *φ* scans were carried out to investigate the in-plane orientation of the SSNO films in regard to LAO substrates by setting the diffraction planes as *ψ* = 45°. As shown in Fig. 1f, fourfold reflection peaks of the film fabricated at 780 °C and 0.2 Pa with an interval of 90° between two adjacent peaks occur at the same angle as that of the LAO substrate, indicating the SSNO films were epitaxially grown on LAO(001) substrates with a cube-on-cube orientation.

**Fig. 2:**
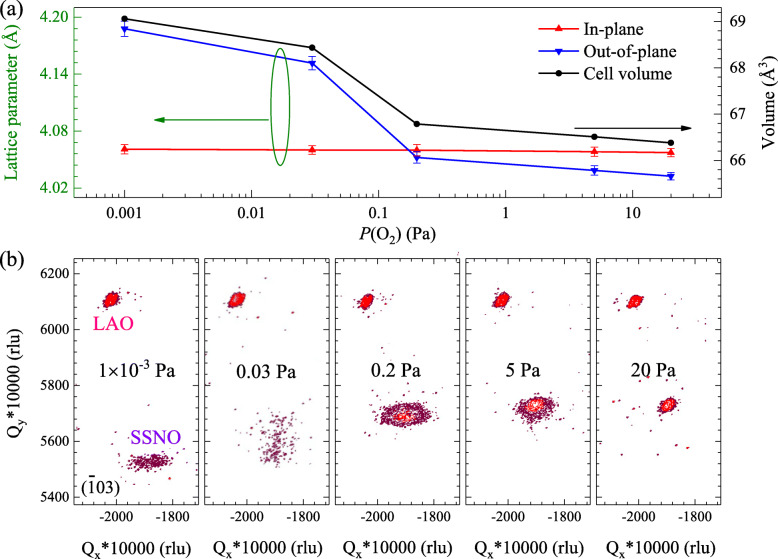
**a** Variations of in-plane and out-of-plane lattice parameters and cell volume with increasing oxygen pressure from 1 × 10^−3^ to 20 Pa. **b** XRD RSMs on (1̅03) reflection collected from the SSNO/LAO heterostructures

The in-plane and out-of-plane lattice parameters for the films deposited under various oxygen pressures can be calculated using (002) and (101) diffraction peaks from Fig. 1a and b. As shown in Fig. 2a, the cell volume and the lattice parameters exhibit the same decreasing trend with increasing oxygen pressure from 1 × 10^−3^ to 20 Pa. The calculated values of the three parameters for these deposited samples are presented in Table 1. The variation in lattice constants with deposition oxygen pressure has also been observed in other oxygen-deficient perovskite films [29, 35] and it can be ascribed to the existence of the oxygen vacancies. In fact, there exists strong Coulomb repulsion between A and B cations (Sr and Sn or Nb in this case), and this interaction will be enhanced by a high density of the positively charged oxygen vacancies [29, 37]. With decreasing the deposition oxygen pressure, the in-plane lattice constants vary less than the out-of-plane lattice constants, which is related to the growth process of the films. Similar phenomenon can also be found in oxygen-deficient BSO films [29]. Figure 2b shows the RSM results of the asymmetric ($\overline{1}$03) reflection obtained from the films with various oxygen pressures. Only diffraction spots from SSNO films and LAO substrates can be observed. One can clearly see that the films are nearly fully relaxed due to the considerable lattice mismatch between the film and the substrate. The values of the lattice mismatch are evaluated to be 7.04, 7.07, 7.12, 7.13, 7.15% with varying oxygen pressure from 20 to 1 × 10^−3^ Pa. Meanwhile, a larger distance between the spots of the film and the substrate is obtained, implying the enlargement in lattice parameters for the SSNO films. Moreover, by lowering oxygen pressure, the reflection spot of the SSNO film becomes dispersive and weaker in intensity, which is in good agreement with the results collected from XRD *ω*-rocking curves mentioned above. The lattice constants *a*, *b*, and *c* of the SSNO films or LAO substrates can also be estimated using the *Q*_x_^*^ and *Q*_y_^*^ values (*a = b* = *−λ/*2*Q*_x_^*^ and *c =* 3*λ/*2*Q*_y_^*^). It is found that the calculated lattice constants from the RSMs are consistent with those from the 2*θ*-*ω* linear scans. The RSM results on ($\overline{1}$03) plane of the films deposited at 0.2 Pa with various substrate temperatures are also shown in Fig. S2. Film spots are observed at almost the same positions, indicating the similar lattice parameters of these films.

**Table 1:** Results of in-plane (*a* = *b*) and out-of-plane (*c*) lattice parameters and cell volumes (*V*) of the SSNO thin films deposited under different oxygen pressures at 780 °C

*P*(O_2_) (Pa)	1 × 10^−3^	0.03	0.2	5	20
*a* = *b* (Å)	4.061	4.060	4.060	4.058	4.057
*c* (Å)	4.188	4.152	4.052	4.039	4.032
*V* (Å^3^)	69.06	68.44	66.79	66.51	66.38

**Fig. 3:**
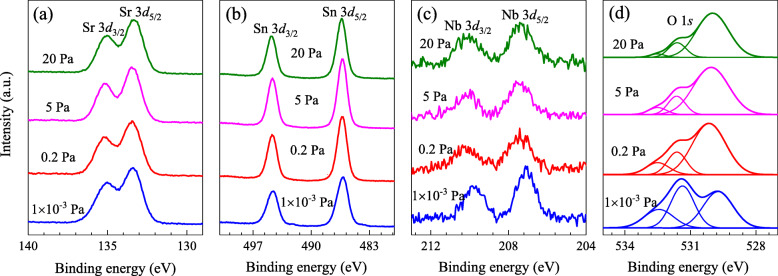
XPS spectra of (**a**) Sr 3*d*, (**b**) Sn 3*d*, (**c**) Nb 3*d*, and (**d**) O 1*s* obtained from the SSNO films with various oxygen pressures

As shown in Fig. 3, XPS was used to investigate the valence states of the chemical elements for the films grown at 780 °C under different oxygen pressures. All the binding energies were corrected by calibrating the C 1*s* peak at 284.6 eV. Figure 3a depicts a doublet of Sr 3*d* spectra with a peak separation of 1.8 eV for all the investigated samples. The binding energies of 135.05 ± 0.10 eV and 133.25 ± 0.10 eV can be assigned to Sr 3*d*_3/2_ and Sr 3*d*_5/2_ lines, respectively, indicating the Sr^2+^ ions in the deposited films [38]. The XPS data of Sn 3*d* states is shown in Fig. 3b. On the basis of NIST database, the binding energies of Sn 3*d*_5/2_ in Sn^0^, Sn^2+^, and Sn^4+^ states are situated approximately at 485.0, 485.9, and 486.6 eV, respectively. For the samples prepared under 20, 5, and 0.2 Pa, the two lines of Sn 3*d*_3/2_ and Sn 3*d*_5/2_ are found to locate at 494.68 and 486.27 eV with a spin-orbit splitting of 8.4 eV, suggesting that only the Sn^4+^ state in these films. However, with decreasing oxygen pressure to 1 × 10^−3^ Pa, the Sn 3*d* peaks shift slightly toward lower binding energy with the positions at 494.59 and 486.18 eV, revealing the partial conversion from Sn^4+^ to Sn^2+^. This result also helps to explain the significant change in lattice constants of the sample as the ionic radius of Sn^2+^ (1.12 Å) is larger than Sn^4+^ (0.69 Å), consequently, a promotion effect on the enlargement of lattice. Similar valence transition phenomenon in Sn can also be observed in Ta-doped SSO films [26] and La-doped BSO films [39]. Figure 3c shows the Nb 3*d* spectra with a doublet correspond to the transitions from Nb 3*d*_3/2_ and Nb 3*d*_5/2_ separated by 2.7 eV. It can be seen that the binding energies of Nb 3*d*_3/2_ and Nb 3*d*_5/2_ appear at about 210.10 and 207.40 eV for the samples at 0.2-20 Pa, while decreasing to 209.77 and 207.07 eV for 1 × 10^−3^ Pa. This result confirms that the Nb ions are presented in +5 state in the SSNO films [40–42]. The slight decrease in binding energies of Nb 3*d* signal for the sample prepared under a high vacuum may be due to the changes in the chemical environment around Nb ions. Figure 3d describes the asymmetric O 1*s* signals of the SSNO films. All of the data can be splitted into three mixed curves employing Gaussian-Lorentzian function. One peak located at the lowest binding energy of 529.94 ± 0.15 eV corresponds to the lattice oxygen, while the other two peaks with higher binding energies of 531.48 ± 0.15 eV and 532.50 ± 0.15 eV are correlated with O^2−^ ions located at oxygen vacancy regions and loosely bound oxygen, respectively [29, 43]. The integrated areas under the low, middle, and high binding energy peaks are denoted as O_A_, O_B_, and O_C_. Then the relative oxygen vacancy concentration for each film is quantified by calculating [O_B_/(O_A_ + O_B_)]. Values of this ratio taken from the data in Fig. 3d are 47.5, 19.8, 16.0, and 15.1% with increasing oxygen pressure from 1 × 10^−3^ to 20 Pa, suggesting that the concentration of oxygen vacancies gradually increases with lowering oxygen pressure in the SSNO films.

**Fig. 4:**
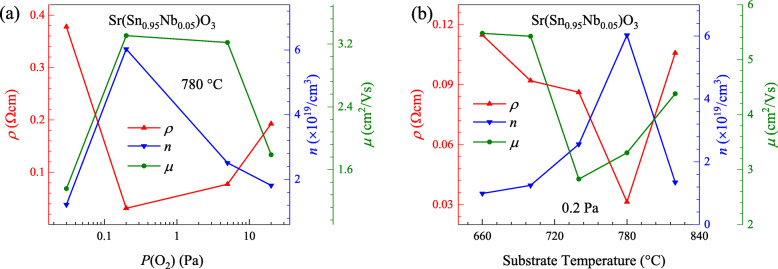
Resistivity, carrier concentration, and mobility of the SSNO films as a function of (**a**) oxygen pressure from 20 to 0.03 Pa and (**b**) substrate temperature from 660 to 820 °C measured at room temperature

To understand the effects of oxygen pressure and deposition temperature on the transport properties of SSNO films, the Hall-effect measurements were performed to determine the carrier concentration (*n*), Hall mobility (*μ*), and electrical resistivity (*ρ*) at room temperature as shown in Fig. 4. The sample at 1 × 10^−3^ Pa was measured to have a high resistivity of ~ 100 MΩ (not shown), and the other films all exhibited *n*-type conduction. As can be seen from Fig. 4a, the carrier concentration increases to 6.03 × 10^19^/cm^3^ with lowering oxygen pressure from 20 to 0.2 Pa. The electrons, as major charge carriers in SSNO films, are produced by the ionization from both oxygen vacancies and the replacement of Sn sites with Nb. The Nb concentration can be estimated from XPS measurements by comparing the areas under the Nb 3*d* and Sn 3*d* peaks and correcting with the sensitivity factors. The atomic ratios of Nb/(Sn + Nb) are calculated to be 0.061, 0.064, and 0.071 for films grown under 0.2, 5, and 20 Pa, respectively. This increase in dopant concentration with increasing oxygen pressure can also be found in Gd-doped ZnO thin films [44]. The calculated Nb concentrations are slightly larger than the nominal doping content in the SSNO films, which may be due to the semi-quantitative XPS analysis. On the other hand, with decreasing oxygen pressure to 0.2 Pa, the relative oxygen vacancy concentration gradually increases as can be proved from XPS results. Therefore, more carriers donated by the increased number of oxygen vacancies, as well as the variation of doping concentration may explain the cause of higher carrier concentration. It should be noted that the shift in the peak position of the (002) peaks with varying the oxygen pressure may also be related to the deviation in the dopant concentration. The electron mobility varies with the same trend of carrier concentration, exhibiting a maximum value of 3.31 cm^2^/Vs at 0.2 Pa. The decreased mobility with increasing oxygen pressure originates from the shift of the Fermi level toward the center of the gap, leading to the greater effectiveness of scattering centers situated below the conduction band edge [45]. In consideration of the relationship of *ρ* = 1/*neμ* (where *e* is the electron charge), the lowest room-temperature resistivity of 31.3 mΩcm observed at 0.2 Pa is the result of the largest carrier concentration and electron mobility at this deposition oxygen pressure. However, with decreasing oxygen pressure to 0.03 Pa, then to 1 × 10^−3^ Pa, considerable oxygen defects are generated in the SSNO films, which possess disordered structure, poor crystallinity (see inset of Fig. 1c) that favor electron localization [46]. Moreover, XPS analysis indicates the charge disproportionation of Sn^2+^ and Sn^4+^ in the sample at 1 × 10^−3^ Pa, which will further block the increase in carrier concentration and suppress the electrical conductivity [41]. Consequently, the significant degradation in transport performance is obtained from the sample at 1 × 10^−3^ Pa. Similar variation tendency has also been observed in ZnO films [47] and BSO films [48]. Further, with the deposition oxygen pressure set at 0.2 Pa, the dependence of conductive performances of the films on substrate temperature was investigated as presented in Fig. 4b. Obviously, the carrier density initially increases gradually as the temperature increases from 660 to 780 °C, and the maximum value is achieved for the 780 °C-deposited sample with a value of 6.03 × 10^19^/cm^3^. The resistivity exhibits an opposite tendency with carrier concentration, indicating that the electrical conductivity of these films is largely determined by the carrier concentration. The lowest room-temperature resistivity of 31.3 mΩcm is obtained at 780 °C. Hall mobility is observed to have nearly the same deposition-temperature relationship with resistivity. Therefore, it is found that the conductivity of the SSNO films can also be tuned by substrate temperature.

**Fig. 5:**
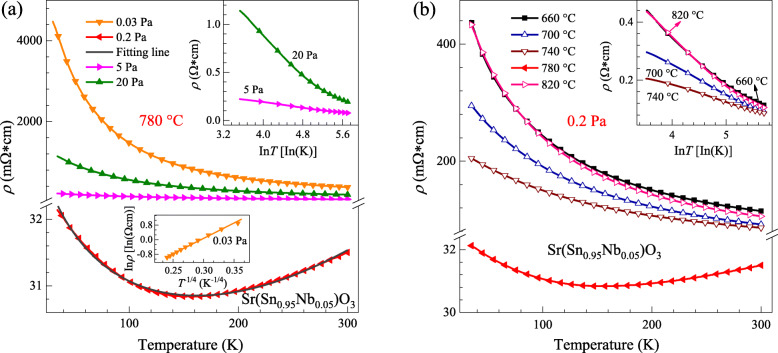
Temperature dependence of resistivity for the SSNO films grown at various oxygen pressures (**a**) and various substrate temperatures (**b**). The upper inset of **a** shows the corresponding linear fit results of ρ vs In*T*, the lower inset the In*ρ* and *T*^−1/4^. The inset of **b** is also the linear relation between *ρ* and In*T*

Figure 5a and b show the temperature-dependent resistivity curves for the SSNO films grown under different conditions within the temperature range of 30-300 K. For the film deposited at 0.03 Pa, the resistivity increases with decreasing temperature (*dρ*/*dT* < 0), which is the characteristic of a semiconducting behavior. To understand the conducting mechanism, a detailed analysis of the temperature-dependent resistivity was carried out. As shown in the lower inset of Fig. 5a, there exists a linear relation between In*ρ* and *T*^−1/4^, suggesting that the variable-range hopping is the dominant conduction mechanism [49]. It can be noticed that only the film deposited at 0.2 Pa with the substrate temperature of 780 °C exhibits a metal-semiconductor transition (MST) at 157 K. The metallic behavior above MST temperature can be attributed to the formation of a degenerate band due to the large density of carriers introduced into the system, while the semiconducting behavior at lower temperatures can be explained by the localization of electrons by disorder [50, 51]. Similar MST behavior can also be found in Ta-doped SSO [26] and oxygen-deficient BSO films [29]. For a further explanation of the transport mechanism in this film, a model of conductivity in disordered matter can be employed when the electronic Fermi wavelength *λ*_*F*_ = [2*π*/(3*π*^2^*n*)^1/3^] and the mean free path *l* = (*h*/*ρne*^2^*λ*_*F*_) become comparable [50, 52, 53]. At low temperatures, *λ*_*F*_ and *l* are estimated to be comparable. Hence, the semiclassical Boltzmann equation of resistivity is taken into consideration to fit the experimental data as described by the following relationship [54–56]:

1 $$ \rho (T)=\kern0.5em \frac{1}{\sigma_0+{a}_1T+{a}_2{T}^{1/2}}\kern0.75em +b{T}^2 $$

where *σ*_0_ is the residual conductivity, *a*_1_*T* term corresponds to the weak localization, and *a*_2_*T*^1/2^ describes the *e*^−^-*e*^−^ interactions for a 3D film. The term *bT*^2^ is included for extended description to scattering contribution at higher temperatures. The solid dark gray line through the experimental data in Fig. 5a suggests that equation (1) gives an excellent fitting result of *ρ*(*T*). Values of the fitting results are *σ*_0_ = 28.0 mΩ^−1^ cm^−1^, *a*_1_ = −0.02 mΩ^−1^cm^−1^K^−1^, *a*_2_ = 0.65 mΩ^−1^cm^−1^K^−1/2^, and *b* = 9.19 × 10^−9^ mΩcmK^−2^, respectively. It can be concluded that the electron to electron interactions are mainly responsible for the contribution to the resistivity at low temperatures as *a*_1_ is much smaller than *a*_2_. With increasing oxygen pressure to 5 and 20 Pa, the resistivity value of the SSNO films increases gradually, and the semiconducting behavior dominates in the whole measured temperature range. As shown in the upper inset of Fig. 5a, a well linear relationship of *ρ*-In*T* curves of the two samples can be observed, indicating that the corresponding mechanism is the two-dimensional weak localization [57], which is essentially caused by quantum-interference of the conduction electrons on the defects of the systems. Considering the inelastic scattering in some electron conduction paths, the interference effect only exists at *t* < *t*_1_ when one electron begins to diffuse from a certain point at *t* = 0. Here, *t*_1_ is the inelastic scattering time. The samples with various substrate temperatures in Fig. 5b all exhibit semiconductor behavior in the temperature range from 30 to 300 K. It is clearly seen that the temperature-dependent resistivity initially decreases from 660 to 780 °C, then increases to 820 °C with the oxygen pressure fixed at 0.2 Pa. As shown in the inset of Fig. 5b, linear relation between *ρ* and In*T* for samples at 660, 700, 740, and 820 °C also indicates the weak localization mechanism [58, 59].

**Fig. 6:**
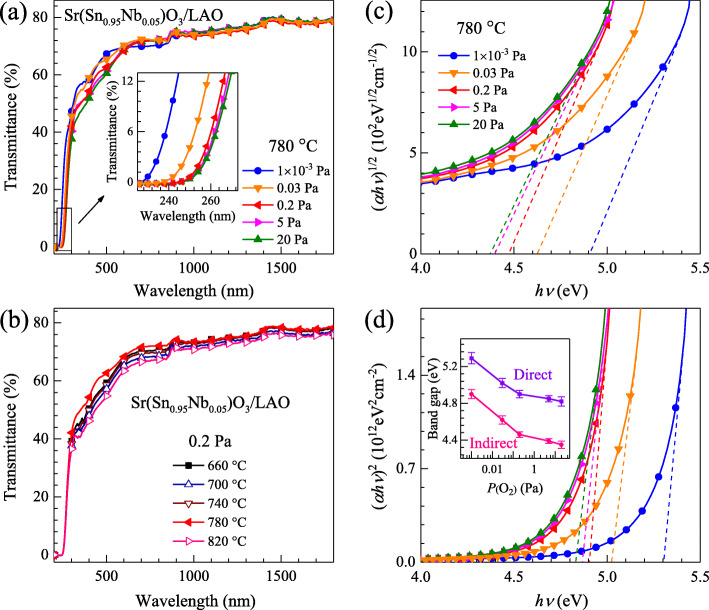
The optical transmittance of the SSNO films fabricated at (**a**) different oxygen pressures and (**b**) different substrate temperatures in the wavelength range of 200-1800 nm. The plots of (*αhν*)^1/2^ versus *hν* and (*αhν*)^2^ versus *hν* for the films with various oxygen pressures are shown in Fig. 6c and d, respectively. The inset in Fig. 6d shows the direct and indirect band-gap energy variations by varying oxygen pressure from 1 × 10^−3^ to 20 Pa

The transmission spectra in the wavelength range of 200-1800 nm for the SSNO films deposited under 1 × 10^−3^-20 Pa and at 660-820 °C are shown in Fig. 6a and b, respectively. Optical transparency of the films with various oxygen pressures and deposition temperatures is more than 70% in the spectral range between 600 and 1800 nm, although the substrate may absorb partial light. The films show a high transmittance up to the near-infrared region, which is required for TCO application in solar cells. This characteristic differs from the decreased transmittance in the near-infrared region due to the absorption for most TCO films [20, 60]. In addition, they are also observed to have the fundamental absorption edges, which lie in the near-ultraviolet region. It is seen from Fig. 6a that the absorption edges of SSNO films deposited at 780 °C shift to shorter wavelength with decreasing oxygen pressure from 20 to 1 × 10^−3^ Pa, as shown more clearly in the inset. However, with the oxygen pressure fixed at 0.2 Pa, the absorption edges of the films grown at various substrate temperatures almost overlapped as seen in Fig. 6b, indicating that the deposition temperature does not obviously modulate the optical properties of the SSNO films. The band gaps *E*_*g*_ of the films can be estimated from the following equation:

2 $$ {\left(\alpha h\nu \right)}^n=A\left( h\nu -{\mathrm{E}}_{\mathrm{g}}\right) $$

where *α* represents the absorption coefficient, *hν* is the photon energy, *n* = 1/2 for indirect band gap and 2 for direct, *A* is a constant related to electron-hole mobility, and *E*_*g*_ is the separation between the bottom of the conduction band and the top of the valence band. Here, *α* can be calculated using the relationship, *α* = (1/*d*)In(1/*T*), where *d* stands for the film thickness and *T* is the transmittance. Figure 6c and d show the plots of (*αhν*)^1/2^ and (*αhν*)^2^ versus *hν* for the samples deposited at 780 °C with different oxygen pressures, respectively. The band gaps can be obtained by extrapolating the linear portions of the curves to *α* = 0. Importantly, the band gap is evaluated to increase with decreasing oxygen pressure from 4.35 to 4.90 eV for the indirect gap and from 4.82 to 5.29 eV for the direct, described in the inset of Fig. 6d. With varying oxygen pressure from 20 to 0.2 Pa, the band gap widening of SSNO film with increased carrier concentration is related to the raised Fermi level in the conduction band of an *n*-type semiconductor, which is referred to as Burstein-Moss effect [61, 62]. As the low energy levels of conduction band were filled up by the conduction electrons, only the photons with higher energies can be absorbed, leading to an enlarged band gap [63]. For the samples deposited at lower oxygen pressures, the further increment in band gap is due to the generation of considerable oxygen vacancies in this film [35].

## Conclusions

In summary, epitaxial Nb-doped SrSnO_3_ thin films under different oxygen pressures and substrate temperatures were fabricated on LAO(001) substrates employing PLD. Film structures were characterized in detail using high-resolution X-ray diffraction, including 2*θ*-*ω* scans, *φ* scans, *ω* scan rocking curves, and RSM. XPS analysis reveals that the Nb^5+^ is present in the SSNO films. Hall-effect measurements were carried out and the sample deposited at 0.2 Pa and 780 °C possesses the lowest room-temperature resistivity of 31.3 mΩcm, with the mobility of 3.31 cm^2^/Vs and carrier concentration of 6.03 × 10^19^/cm^3^. Temperature-dependent resistivity of this film shows a metal-semiconductor transition, which is discussed based on electron-electron interactions. However, the films grown at other conditions all exhibit semiconducting behavior, which can be analyzed using variable-range hopping or the two-dimensional weak localization model. A high optical transmittance of more than 70% for the films is observed in the wavelength range of 600 to 1800 nm. For the films with different oxygen pressures, the variation of band gap is attributed to Burstein-Moss shift and oxygen vacancies. The SSNO film can be tuned flexibly between an insulator and a conductor just by varying the oxygen deposition pressure. Such characteristic can be used in field-effect transistors and other electronic devices consisting of both insulating dielectric and conducting electrodes.

## Supplementary information

**Additional file 1: Fig. S1.** Wide-angle XRD 2*θ*-*ω* scan results of the SSNO (top) and SSO films (bottom) deposited at 780 °C and 20 Pa. **Fig. S2.** RSMs around (1̅03) reflections for the films with various substrate temperatures from 660 to 820 °C.

## Data Availability

The datasets and supporting information obtained in this paper are included in this article.

## Abbreviations

TCO: Transparent conducting oxides
MST: Metal-semiconductor transition
FWHM: Full width at half maximum
