# Supplementary material for: Electrical and Optical Properties of Nb-doped SrSnO3 Epitaxial Films Deposited by Pulsed Laser Deposition
Source: Nanoscale Res Lett. 2020 Aug 17;15:164. doi: 10.1186/s11671-020-03390-1 (PMC7431481; doi:10.1186/s11671-020-03390-1)
Supplement: Supplementary file 1 — Additional file 1: Fig. S1. Wide-angle XRD 2θ-ω scan results of the SSNO (top) and SSO films (bottom) deposited at 780 °C and 20 Pa. Fig. S2. RSMs around (1̅03) reflections for the films with various substrate temperatures from 660 to 820 °C. [file 11671_2020_3390_MOESM1_ESM.pdf]

Supplementary Materials

Electrical and Optical Properties of Nb-doped SrSnO3 Epitaxial Films Deposited by Pulsed Laser Deposition

Kaifeng Li[1], Qiang Gao[1], Li Zhao[1], Qinzhuang Liu[1,2*]

[1]*School of Physics and Electronic Information, Huaibei Normal University, Huaibei 235000, P. R. China*

[2]*Anhui Province Key Laboratory of Pollutant Sensitive Materials and Environmental Remediation, Huaibei Normal University, Huaibei 235000, P. R. China*

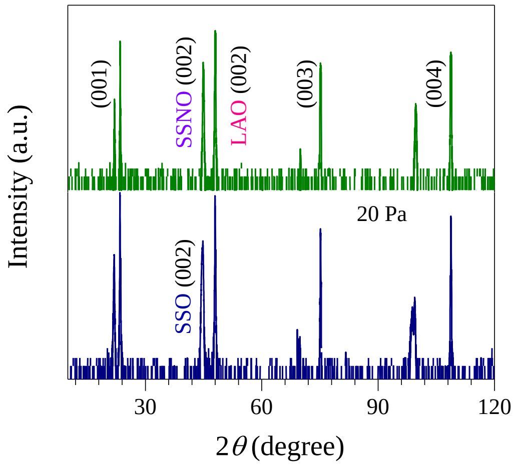

Fig. S1 Wide-angle XRD 2θ-ω scan results of the SSNO (top) and SSO films (bottom) deposited at 780 °C and 20 Pa.

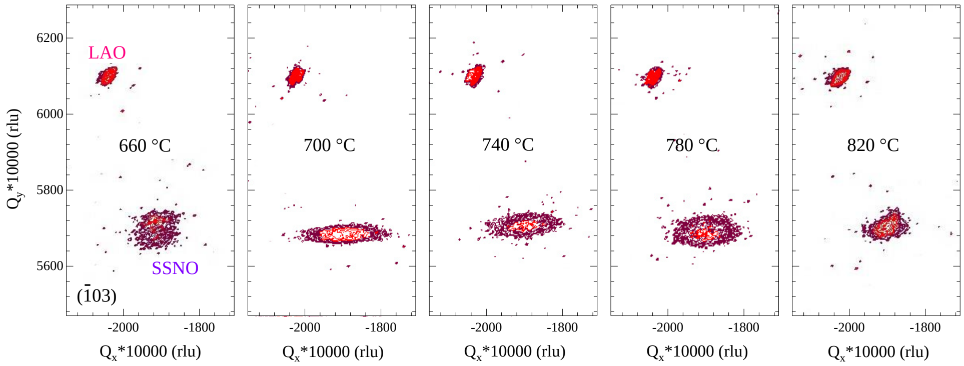

Fig. S2 RSMs around ($\bar{1}03$) reflections for the films with various substrate temperatures from 660 to 820 °C.

*Corresponding Author E-mail:qzliu@mail.ustc.edu.cn
